# Long-term weight loss outcome of laparoscopic Roux-en-Y gastric bypass predicted by weight loss at 6 months in Chinese patients with BMI ≥ 32.5 kg/m^2^

**DOI:** 10.1097/MD.0000000000033235

**Published:** 2023-03-24

**Authors:** Qiqige Wuyun, Dezhong Wang, Chenxu Tian, Guangzhong Xu, Buhe Amin, Dongbo Lian, Dexiao Du, Weihua Zhang, Min Jiang, Guanyang Chen, Nengwei Zhang, Liang Wang

**Affiliations:** a Surgery Centre of Diabetes Mellitus, Capital Medical University Affiliated Beijing Shijitan Hospital, Beijing, China; b General Surgery; Aerospace Center Hospital, Beijing, China.

**Keywords:** BMI ≥ 32.5 kg/m^2^, laparoscopic Roux-en-Y gastric bypass, prognostic prediction, weight loss

## Abstract

Laparoscopic Roux-en-Y gastric bypass (LRYGB) is classic bariatric procedure with long-term safety and efficacy. However, no studies have focused on predicting long-term weight loss after LRYGB in Chinese patients with body mass index (BMI) ≥ 32.5 kg/m^2^. To explore the relationship between initial and long-term weight loss after LRYGB in patients with BMI ≥ 32.5 kg/m^2^. All patients were followed-up to evaluate BMI, percentage of excess weight loss (%EWL), and comorbidities. Linear and logistic regression were performed to assess the relationship between initial and long-term weight loss. Receiver operating characteristic curve was used to determine optimal cutoff value. We enrolled 104 patients. The median preoperative BMI was 41.44 (37.92–47.53) kg/m^2^. %EWL ≥ 50% at 5 years was considered as successful weight loss, and 75.00% of the patients successfully lost weight. The cure rates of hypertension, hyperlipidemia, and type 2 diabetes mellitus at 1 year were 84.38%, 33.93%, and 60.82%, respectively. %EWL at 6 months and 5 years were positively correlated and its relationship could be described by following linear equation: %EWL_5 years_ = 43.934 + 0.356 × %EWL_6 months_ (*P* < .001; r^2^ = 0.166). The best cutoff %EWL at 6 months after LRYGB to predict 5-year successful weight loss was 63.93% (sensitivity, 53.85%; specificity, 84.62%; area under the curve (AUC) = 0.671). In Chinese patients with BMI ≥ 32.5 kg/m^2^, %EWL at 6 months and 5 years were positively correlated and %EWL at 5 years could be calculated by following linear equation: %EWL_5 years_ = 43.934 + 0.356 × %EWL_6 months_.

## 1. Introduction

Laparoscopic Roux-en-Y gastric bypass (LRYGB) is a surgical procedure conventionally performed to treat obesity-related complications while reducing the patient’s weight. It has reliable efficacy in patients with type 2 diabetes mellitus (T2DM).^[1–7]^

Although the number of laparoscopic sleeve gastrectomy (LSG) procedures is increasing every year and LSG is accounting for an increasing proportion of all bariatric procedures, LRYGB is widely recommended for patients with body mass index (BMI) ≥ 40 or ≥ 35 kg/m^2^ with severe obesity-related comorbidities, particularly T2DM.^[5]^

Initial weight loss indicators are used to predict weight loss after LSG.^[8–11]^ Timely prediction of patients who are unlikely to maintain long-term weight loss can help design more targeted lifestyle, medical, and behavioral interventions as early as possible to improve the prognosis and increase surgical satisfaction of these patients. However, in Chinese patients with BMI ≥ 32.5 kg/m^2^ undergoing LRYGB, weight loss at 5 years is challenging to predict.

The aim of the present study was to explore the association between initial weight loss indicators after LRYGB and weight loss at 5 years in Chinese patients with BMI ≥ 32.5 kg/m^2^ and establish a predictive model.

## 2. Methods

### 2.1. Inclusion and exclusion criteria

In this retrospective study, we enrolled 104 patients who underwent LRYGB at our hospital from October 2014 to April 2017.

#### 1.2.1. Inclusion criteria were.

1. Treatment of obesity with LRYGB.
2. Preoperative BMI ≥ 32.5 kg/m2; and
3. Chinese residence.

#### 12.2. Exclusion criteria were.

1. Lack of 5-year postoperative follow-up results; and
2. Development of comorbidities, such as cancer and psychiatric disorders, during the 5-year follow-up period.

The Ethics Review Board of our hospital approved the study protocol (sjtky11-1x-2022 (076)). Written informed consent was obtained from all participants. All procedures performed in studies involving human participants were in accordance with the ethical standards of the 1964 Helsinki declaration and its later amendments or comparable ethical standards.

### 2.2. Definitions of relative terms

Based on our previous reports,^[8,12]^ the diagnostic criteria and curative outcome were defined for comorbidities, including T2DM, hypertension, and hyperlipidemia.

Hypertension in these patients was defined as SBP ≥ 140 mm Hg or DBP ≥ 90 mm Hg, and remission was defined by blood pressure values below these thresholds without the use of antihypertensive medication at 1-year post-LRYGB. T2DM was defined by a hemoglobin A1c level of ≥ 6.5%, with remission being defined by an hemoglobin A1c of < 6.0% without the use of insulin or oral hypoglycemic drugs at 1-year post-LSG. Lipid profiles were analyzed by the determination of fasting total cholesterol and triglyceride levels, together with the cholesterol to high-density lipoprotein ratios. Hyperlipidemic remission was defined by the reduction of these values to fall within normal ranges at 1-year post-LRYGB without the need for lipid-lowering drugs. Postoperative percentage of excess weight loss (%EWL) ≥ 50% was regarded as weight loss success, as is common. The long-term weight loss outcome was defined as the weight loss outcome at 5 years postoperatively.

### 2.3. Operative techniques and postoperative follow-up

The operative techniques and postoperative follow-up were described in our previous studies.^[8,12]^ Three surgeons performed all LRYGB procedures in enrolled patients using a standardized approach. We first constructed a small pouch (approximately 20 mL volume) at the proximal end of the stomach, and the fundus of the stomach was completely isolated. We then transected the proximal jejunum 70 to 120 cm from the Treitz ligament. After anastomosis of the distal end of the jejunum to the small gastric pouch, the afferent biliopancreatic limb was anastomosed to the jejunum 100 to 150 cm distally. The total length of the biliopancreatic branch and the food branch was approximately 250-cm.

Upper gastrointestinal contrast studies were conducted postoperatively on day 1 to 3. When no abnormalities were detected, patients were allowed to freely consume water and were given a liquid diet. The patients can be discharged without any symptoms such as vomiting. The follow-up was completed via phone or WeChat for outpatients and at the hospital for inpatients. Follow-up at 3, 6, 12, 36, and 60 months after LRYGB to assess various physiological parameters of the patients.

### 2.4. Statistical analysis

Nominal data were analyzed using the Fisher exact or chi-squared test. Normally distributed data are expressed as mean ± standard deviation, while non-normally distributed are expressed as median (interquartile range). The independent-samples *t* test and nonparametric test were performed to compare normally and non-normally distributed data, respectively. Based on initial weight loss, linear and binary logistic regression analyses were employed to evaluate differences in weight loss obtained 5 years after LRYGB. The receiver operating characteristic (ROC) curve was used to determine the optimal cutoff values for initial weight loss indicators. A 2-sided *P* value < .05 was considered to indicate statistical significance. ROC curves were drawn using MedCalc version 19.2.6 (MedCalc, Inc., Mariakerke, Belgium). Histograms were plotted using Graphpad Prism version 8.4.3 (GraphPad Software, San Diego, CA). Other statistical tests were performed using SPSS version 20.0 (IBM Corp., Armonk, NY).

## 3. Results

We enrolled a total of 104 patients with age and preoperative BMI of 41.71 ± 11.87 years and 41.44 (37.92–47.53) kg/m^2^, respectively. Preoperatively, 93.27% of the patients had T2DM. Table 1 lists the preoperative patient’s characteristics.

**Table 1:** The preoperative patients characteristics.

Variable	Value
Age (yr)	41.71 ± 11.87 *
Gender	
Male (%)	51 (49.04%) †
Female (%)	53 (50.96%)
The ethnic	
Han (%)	98 (94.23%)
Other ethnics (%)	6 (5.77%)
Residence	
Urban (%)	78 (75.00%)
Rural (%)	26 (25.00%)
North & South	
North (%)	94 (90.38%)
South (%)	10 (9.62%)
Preoperative BMI (kg/m^2^)	41.44 (37.92, 47.53) ‡
T2DM (%)	97 (93.27%)
Hypertension (%)	32 (30.77%)
Hyperlipidemia (%)	56 (53.85%)
Hyperuricemia (%)	79 (75.96%)
Fatty liver (%)	96 (92.31%)
OSA (%)	104 (100.00%)
PCOS (%)	31 (58.49%)

BMI = body mass index, OSA = obstructive sleep apnea, PCOS = polycystic ovary syndrome, T2DM = type 2 diabetes mellitus.

* Mean ± SD,

† No. (%),

‡ Median (upper and lower quartiles).

**Figure 1.**
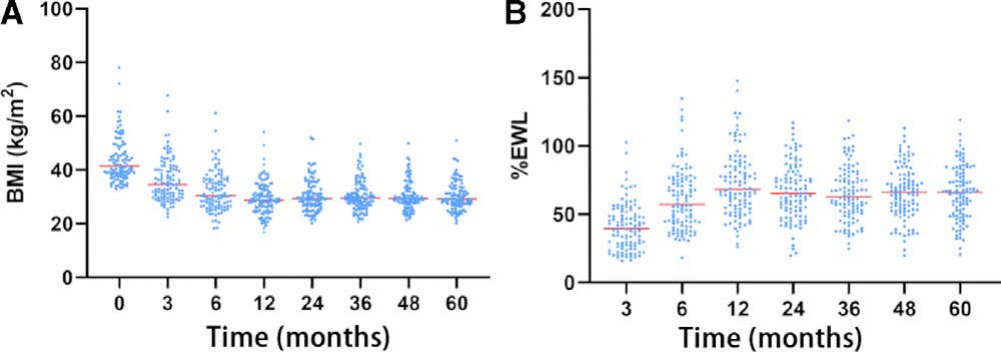
Trends in the BMI (A) and %EWL (B) over time. (Note: Each point in the figure represents a patient, and the red horizontal line represents the median). %EWL = percentage of excess weight loss.

None of the patients developed serious postoperative complications, such as death, fistula, stenosis, or bleeding. The remission rates of hypertension, hyperlipidemia, and T2DM 1 year postoperatively were 84.38%, 33.93%, and 90.77%, respectively. The preoperative and 3- and 6-month and 1-, 2-, 3-, 4-, and 5-year postoperative BMIs were 41.44 (37.92–47.53), 34.58 (29.93–40.25), 30.44 (26.58–36.40), 28.78 (25.13–32.87), 29.28 (26.58–33.76), 29.62 (26.88–33.06), 29.37 (27.35–32.95), and 29.19 (27.10–33.36) kg/m^2^, respectively. The %EWLs at 3 and 6 months and 1, 2, 3, 4, and 5 years were 39.51 (27.08–52.00), 57.22 (43.46–75.39), 68.20 (54.21–85.42), 65.37 (52.46–78.90), 62.98 (51.69–78.06), 66.14 (53.26–78.79), and 66.15 (50.17–80.19), respectively. Figure 1 shows the trends in BMI and %EWL over time. At 5 years, 78 (75.00%) patients achieved successful weight loss, whereas 26 (25.00%) patients did not. Weight loss at 5 years was 38.93 (12.5–121.6) kg.

Table 2 compares the baseline data between patients with and without successful weight loss. Baseline data did not differ significantly between patients with and without successful weight loss. The binary logistic regression analysis performed with successful weight loss at 5 years as the dependent variable and %EWLs at 3 and 6 months as the independent variables showed that only the 6-month postoperative %EWL was a statistically significant independent variable (Table 3). Therefore, 6-month postoperative %EWL was used for the predictive analysis.

**Table 2:** The comparison of the baseline data between successful and fail weight loss groups at 5 years.

	Successful weight loss group (n = 78)	Failed weight loss group (n = 26)	*x ^2^/ z/ t*	*P* value
Age (yr)	41.45 ± 11.70 *	42.50 ± 12.57	0.390	.698
Gender				
Male (%)	40 (51.28%) †	11 (42.31%)	0.628	.428
Female (%)	38 (48.72%)	15 (57.69%)		
The ethnic				
Han (%)	74 (94.87%)	24 (92.31%)	–	.638
Other ethnics (%)	4 (5.13%)	2 (7.69%)		
Residence				
Urban (%)	59 (75.64%)	19 (73.08%)	0.068	.794
Rural (%)	19 (24.36%)	7 (26.92%)		
North & South				
North (%)	8 (10.26%)	2 (7.69%)	–	1.000
South (%)	70 (89.74%)	24 (92.31%)		
Preoperative BMI (kg/m^2^)	40.77 (37.20, 44.10) ‡	43.48 (39.13, 50.19)	−1.329	.184
T2DM (%)	73 (96.05%)	24 (92.31%)	-	1.000
Hypertension (%)	21 (26.92%)	11 (42.31%)	2.167	.141
Hyperlipidemia (%)	39 (50.00%)	17 (65.38%)	1.857	.173
Hyperuricemia (%)	57 (73.08%)	22 (84.62%)	1.422	.233
Fatty liver (%)	71 (91.03%)	25 (96.15%)	–	.676
OSA (%)	78 (100.00%)	26 (100%)	–	1.000
PCOS (%)	21 (55.26%)	10 (66.67%)	0.576	.448

BMI = body mass index, OSA = obstructive sleep apnea, PCOS = polycystic ovary syndrome, T2DM = type 2 diabetes mellitus.

* Mean ± SD,

† No. (%),

‡ Median (upper and lower quartiles).

**Table 3:** Logistic regression analysis of factors for successful weight loss.

	OR (95% CI)	*P* value
%EWL at 3 months	0.970 (0.929, 1.013) *	.173
%EWL at 6 months	1.046 (1.007, 1.086)	.019

%EWL = percentage of excess weight loss.

* Median (upper and lower quartiles).

**Figure 2.**
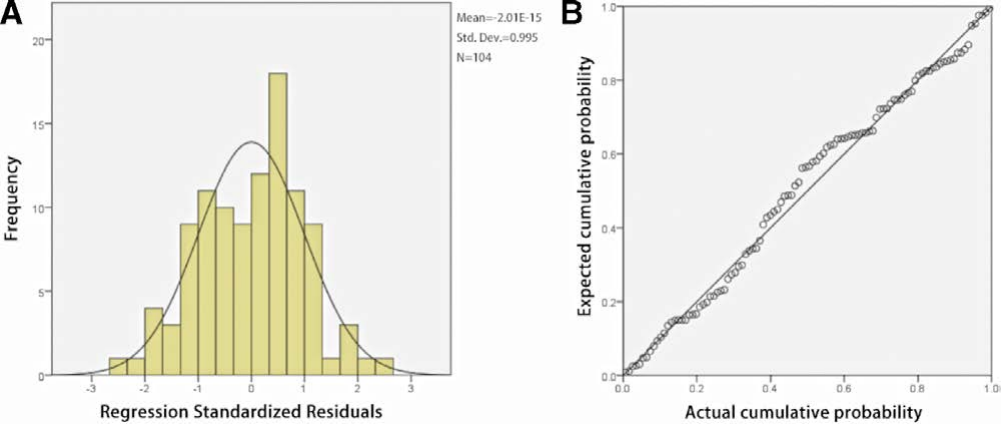
Histograms (A) and probability–probability plots (B) of standardized residuals for %EWL at 5 years. %EWL = percentage of excess weight loss.

To further clarify the relationship between %EWL at 6 months and long-term weight loss, the linear regression model developed using %EWL at 5 years as the dependent variable and %EWL at 6 months as the independent variable showed a significant positive correlation between %EWLs at 5 years and 6 months (*P* < .001; r^2^ = 0.166). Figure 2 shows the histograms and probability–probability plots of standardized residuals. The following linear equation explained the relationship between initial and long-term postoperative weight loss:

%EWL_5 years_ = 43.934 + 0.356 × %EWL_6 months_

**Figure 3.**
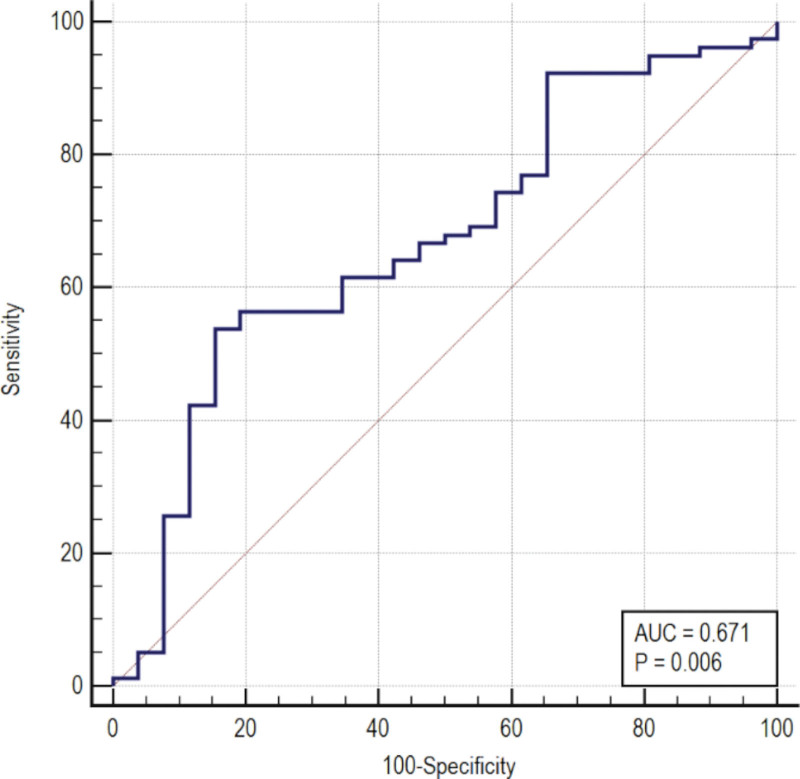
ROC curve of %EWL at 6 months among patients with successful %EWL at 5 years after LRYGB. %EWL = percentage of excess weight loss, LRYGB = laparoscopic Roux-en-Y gastric bypass, ROC = receiver operating characteristic.

Postoperative %EWL ≥ 50% was regarded as weight loss success. Weight loss success at 5 years was the categorical variable, and %EWL at 6 months was the variable used to draw the ROC curves. Figure 3 shows the results. The optimal cutoff %EWL at 6 months for predicting the weight loss outcome at 5 years was 63.93% (area under the ROC curve, 0.671; 95% confidence interval: 0.572–0.760; sensitivity, 53.85%; specificity, 84.62%; *P**** ***< .001).

## 4. Discussion

In this study, the weight loss success rates at 3, 4, and 5 years after LRYGB were 78.85%, 80.77%, and 75%, respectively, and the corresponding BMIs were 29.62 (26.88–33.06), 29.37 (27.35–32.95), and 29.19 (27.10–33.36) kg/m^2^, respectively. The weight loss success rate suggested that the efficacy of LRYGB was satisfactory. However, only 17 (16.35%) patients had a BMI below the diagnostic criteria for overweight, that is, 25 kg/m^2^. From this viewpoint, the weight loss seemed to be less than ideal. However, discussing the postoperative BMI alone is inaccurate. The preoperative BMI of the patients in this study exceeded 32.5 kg/m^2^ at 41.44 (37.92–47.53) kg/m^2^, and the prevalence rates of T2DM, fatty liver, and obstructive sleep apnea were 93.27%, 92.31%, and 100%, respectively. The patients in this study had morbid obesity, which is more difficult to reduce to normal BMI. Therefore, although only 16.35% of the patients could reduce their BMI to below the overweight criteria, LRYGB could be considered to have a good effect. In many large-scale randomized controlled trials, LRYGB was superior or comparable to LSG in terms of weight loss and management of T2DM.^[1,2,13–19]^ Thus, LRYGB could positively affect weight loss in Chinese patients with BMI ≥ 32.5 kg/m^2^.

In this study, the prevalence rates of T2DM, hypertension, and hyperlipidemia were 93.27%, 30.77%, and 53.85%, respectively. Obesity-related comorbidities should be treated while reducing weight loss. The remission rates of hypertension, hyperlipidemia, and T2DM 1 year postoperatively were 84.38%, 33.93%, and 90.77%, respectively. Overall, the cure rate of comorbidities with LRYGB was satisfactory. The aforementioned cure rates suggested that LRYGB was significantly effective in treating obesity-related comorbidities. In the past 2 years of clinical practice at our hospital, T2DM was easier to cure in patients with higher preoperative BMI than in those with lower preoperative BMI using surgery, consistent with recent reports.^[20,21]^ Therefore, for patients with low preoperative BMI and T2DM, we have recently gradually adjusted the recommended strategy for bariatric procedures to LRYGB. Du et al^[22]^ reported that the cure rate of T2DM was 80% in patients who underwent LRYGB with preoperative BMI between 27.5 and 32.5 kg/m^2^. This cure rate of T2DM was less than that in the present study, suggesting that patients with T2DM with low preoperative BMI should undergo LRYGB, which is more effective for T2DM.

In addition, %EWL at 6 months and 5 years were positively correlated (r^2^ = 0.166), and the relationship could be expressed by the following linear equation: %EWL_5 years_ = 43.934 + 0.356 × %EWL_6 months_. The prediction effect of the linear regression equation was acceptable. The ROC curve analysis revealed that the optimal cutoff %EWL at 6 months for predicting the weight loss outcome at 5 years was 63.93% (area under the ROC curve, 0.671; sensitivity, 53.85%; specificity, 84.62%). Although the prediction effect of the ROC curve was unsatisfactory, that of the linear equation was good.

In this study, although the prediction model of the ROC curve was meaningful, it had poor stability, low Youden index, and limited predictive value. There may be 3 reasons. First, the sample size of this study was small, with only 104 cases, which might be insufficient to build a predictive model with a higher Youden index. Second, the total length of the small intestine differed significantly among individuals. After LRYGB was uniformly placed in a 250-cm intestine, the common channel length differed significantly among individuals. This might have resulted in variable weight loss after LRYGB. Third, the size of the gastric pouch constructed by LRYGB was approximately 20 mL, but the gastric pouch size differed among individuals. Further, weight loss in patients with large gastric pouches might be weaker compared to those with small gastric pouches. At 5 years, the specificity of the ROC curve for predicting weight loss success was 84.62%. Thus, the predictive value of the ROC curve analysis of %EWL at 6 months to predict successful weight loss at 5 years was satisfactory.

We tend to recommend bariatric procedures to patients at our hospital. First, considering that LRYGB has a more definite effect on T2DM, we recommend LRYGB for patients with T2DM. Second, we strongly recommend LRYGB for patients with a history of T2DM ≥ 5 years, age ≥ 50 years, and poor preoperative pancreatic islet function (fasting C-peptide < 2.0 ng/mL and 2-hour C-peptide not exceedingly thrice the fasting C-peptide level). Furthermore, for patients with a family history of gastric cancer or *H pylori*-positive or atrophic gastritis, we strongly recommend LSG to prevent carcinogenesis in the stomach. Finally, we recommend LRYGB for patients with ≥ 3 obesity-related comorbidities and LSG for patients with preoperative BMI < 32.5 kg/m^2^ and T2DM only. We thoroughly educate the patients preoperatively, carefully explain the advantages and disadvantages of each bariatric procedure, and share our recommendations. Subsequently, the patients make the decision themselves.

This study had some limitations. This study is a retrospective study, and selective bias is inevitable. First, we followed-up with 104 Chinese patients with BMI ≥ 32.5 kg/m^2^ who underwent LRYGB for 5 years. A model for predicting long-term weight loss was obtained by analyzing the follow-up results. However, it was suggested that after LRYGB, the inter-individual differences in the common channel length and gastric pouch size might directly or indirectly affect long-term weight loss. The common channel length and gastric pouch size were large, and confounding factors were difficult to quantify when building predictive models. Second, we only explored the cure rates of obesity-related comorbidities 1 year postoperatively. Whether or not the patients T2DM, hypertension, or hyperlipidemia recurred after 1 year remains unknown.

## 5. Conclusion

In Chinese patients with BMI ≥ 32.5 kg/m^2^ who underwent LRYGB, %EWL at 6 months was significantly positively correlated with %EWL at 5 years. The relationship could be expressed by the following linear equation: %EWL_5 years_ = 43.934 + 0.356 × %EWL_6 months_.

## Acknowledgments

The authors would like to thank all the reviewers who participated in the article, as well as MJEditor (www.mjeditor.com) for providing English editing services during the preparation of this manuscript.

## Author contributions

**Conceptualization:** Qiqige Wuyun, Guangzhong Xu, Nengwei Zhang, Liang Wang.

**Data curation:** Chenxu Tian, Nengwei Zhang, Liang Wang.

**Formal analysis:** Qiqige Wuyun, Chenxu Tian.

**Funding acquisition:** Weihua Zhang, Min Jiang.

**Methodology:** Dezhong Wang, Dongbo Lian, Nengwei Zhang, Liang Wang.

**Project administration:** Buhe Amin, Guanyang Chen.

**Resources:** Qiqige Wuyun, Guangzhong Xu, Dexiao Du, Nengwei Zhang.

**Software:** Dexiao Du.

**Validation:** Dezhong Wang.

**Visualization:** Dezhong Wang.

**Writing – original draft:** Qiqige Wuyun, Chenxu Tian.

**Writing – review & editing:** Nengwei Zhang, Liang Wang.
